# *HER2*阳性非小细胞肺癌诊断及治疗进展

**DOI:** 10.3779/j.issn.1009-3419.2023.102.14

**Published:** 2023-04-20

**Authors:** REN Chenyi, CAO He, ZHENG Jing, SUN Wenjia, ZHOU Jianya

**Affiliations:** ^1^322000 义乌，浙江大学医学院附属第四医院呼吸与危重症医学科（任晨怡）; ^1^Department of Respiratory Disease, The Fourth Affiliated Hospital, Zhejiang University School of Medicine, Yiwu 322000, China; ^2^310000 杭州，浙江大学医学院附属第一医院呼吸与危重症医学科（曹赫，郑静，孙文佳，周建娅）; ^2^Department of Respiratory Disease, Thoracic Disease Center, The First Affiliated Hospital, Zhejiang University School of Medicine, Hangzhou 310000, China

**Keywords:** 肺肿瘤, 人表皮生长因子受体2, 靶向治疗, Lung neoplasms, Human epidermal growth factor receptor 2, Targeted therapy

## Abstract

肺癌是全球最常见的恶性肿瘤，也是癌症死亡的主要原因。人表皮生长因子受体2（human epidermal growth factor receptor 2, HER2）阳性非小细胞肺癌（non-small cell lung cancer, NSCLC）是指因HER2基因发生突变、扩增、过表达，导致其功能失调，从而发生的NSCLC。HER2是HER家族中活性最高的受体，可以与其他成员结合形成二聚体，激活多种信号通路，从而调节细胞增殖、分化、迁移和凋亡。在NSCLC中，HER2阳性通常被认为是预后不良的标志。目前HER2阳性NSCLC的诊疗尚未成熟。通常使用免疫组化（immunohistochemistry, IHC）、下一代测序（next generation sequencing, NGS）等方法来检测HER2突变、扩增、过表达这些阳性状态。在先前的研究中，抗肿瘤药物在HER2阳性NSCLC中并未显示出理想的疗效。但近些年来，相关研究表明靶向治疗中的抗体药物偶联物（antibody-drug conjugates, ADCs）和新型酪氨酸激酶抑制剂（tyrosine kinase inhibitors, TKIs）对HER2阳性NSCLC显示出较好的抗肿瘤活性。本文归纳了HER2阳性NSCLC的诊断和治疗进展，为后续研究提供参考。

## Abstract (en)

Lung cancer is the most common malignancy in the world and the leading cause of cancer death. Human epidermal growth factor receptor 2 (HER2) positive non-small cell lung cancer (NSCLC) refers to the NSCLC caused by mutation, amplification or overexpression of the HER2 gene, resulting in its dysfunction. HER2 is the most active receptor in the HER family and can combine with other members to form dimers, which can activate multiple signaling pathways and regulate cell proliferation, differentiation, migration and apoptosis. In NSCLC, HER2 positivity is usually considered a poor prognostic marker. At present, the diagnosis and treatment of HER2-positive NSCLC are not mature. Immunohistochemistry (IHC), next generation sequencing (NGS) and other technologies are often used to detect the positive status of HER2 mutation, amplification or overexpression. In previous studies, antitumor drugs did not show ideal therapeutic effects in HER2-positive NSCLC. However, in recent years, related researches have shown that antibody-drug conjugates (ADCs) and new tyrosine kinase inhibitors (TKIs) in targeted therapy show good antitumor activity against HER2 positive NSCLC. This article summarized the progress in diagnosis and treatment of HER2-positive NSCLC, so as to provide reference for subsequent researches.

肺癌是全球最常见的恶性肿瘤，也是癌症死亡的主要原因，非小细胞肺癌（non-small cell lung cancer, NSCLC）占所有肺癌的80%以上^[1]^。针对NSCLC驱动基因的个体化治疗，大大改善了患者的预后。但仍有许多驱动基因阳性的NSCLC的个体化治疗需要进一步研究。因人表皮生长因子受体2（human epidermal growth factor receptor 2, HER2）发生突变、扩增、过表达而引起的NSCLC，可被称为HER2阳性NSCLC。目前，关于HER2阳性状态的检测手段非常多样，这些检测手段的不断发展也在一定程度上促进了HER2阳性NSCLC的治疗研究。相关研究^[2]^表明，与免疫检查点抑制剂（immune checkpoint inhibitors, ICIs）、单克隆抗体等药物相比，新型酪氨酸激酶抑制剂（tyrosine kinase inhibitors, TKIs）及抗体药物偶联物（antibody-drug conjugates, ADCs）在HER2阳性NSCLC中具有更好的抗肿瘤活性。目前关于HER2阳性NSCLC的诊断及治疗仍需探索，本篇文章对HER2阳性NSCLC的诊断及治疗进展进行了相关综述，以期为后续研究提供参考。

## 1 HER2与NSCLC

HER2由三个结构域组成：胞外结构域（extracellular domain, ECD）、跨膜结构域（transmembrane domain, TMD）和胞内酪氨酸激酶结构域（intracellular tyrosine kinase domain, TKD）。HER2位于17号染色体长臂，与HER1、HER3以及HER4一起组成了HER家族，编码跨膜酪氨酸激酶受体蛋白。HER2是该家族中活性最高的受体，没有已知的配体，但可以结合HER家族的其他成员形成异源二聚体，或在高表达时形成同源二聚体，从而激活多种信号通路，包括丝裂原活化蛋白激酶（mitogen-activated protein kinase, MAPK）、磷脂酰肌醇3-激酶（phosphatidylinositol 3-kinase, PI3K）/蛋白激酶B（protein kinase B, AKT）和蛋白酪氨酸激酶-信号转导子及转录激活因子（Janus kinase-signal transducer and activator of transcription, JAK-STAT）信号通路，从而调节细胞增殖、分化、迁移和凋亡^[3,4]^。由此可见，HER2在细胞信号转导中起着关键作用，当其功能失调时癌症很有可能发生。HER2阳性NSCLC常见于女性和非吸烟者，且相比其他驱动基因阳性的NSCLC，其预后较差。HER2阳性也被认为是表皮生长因子受体（epidermal growth factor receptor, EGFR）-TKIs获得性耐药的可能机制^[5]^。HER2突变发生在2%-4% NSCLC患者中，其中以TKD内的外显子20插入突变最常见。HER2扩增与HER2突变几乎不同时发生。相较于乳腺癌，NSCLC中的HER2扩增并不常见，占2%-4%。2.5%-34%的NSCLC患者发生HER2过表达，HER2过表达发生率的范围之所以这么广泛，可能是因为评估方法不一致^[5⇓⇓-8]^。

## 2 HER2阳性NSCLC的诊断进展

NSCLC中HER2阳性状态包括HER2突变、扩增及过表达，不同的HER2阳性状态可能具有不同的治疗方法和预后。选择合适的检测方法，正确诊断HER2阳性状态，对其治疗至关重要。本篇综述对NSCLC中HER2阳性状态的诊断进展进行了阐述，并在表1中罗列了近年来HER2阳性NSCLC研究中的检测方法^[2,9⇓⇓⇓⇓⇓⇓⇓⇓⇓⇓⇓⇓⇓⇓⇓⇓⇓⇓⇓⇓⇓⇓⇓⇓⇓⇓⇓⇓⇓⇓⇓⇓⇓-43]^。

**表1:** HER2阳性NSCLC研究中的检测方法

Author	HER2 status	n	Method
Li et al.^[2]^	HER2 mutation	91	NGS, Oncomine Dx Target test
Yang et al.^[9]^	HER2 mutation	198	NGS
	HER2 amplification	13	NGS (HER2 copy number≥3.62)
Zhou et al.^[10]^	HER2 mutation	44	ARMS-PCR, NGS
Wang et al.^[11]^	HER2 mutation	25	ARMS-PCR
Saalfeld et al.^[12]^	HER2 mutation	61	Gene panel sequencing
Guisier et al.^[13]^	HER2 mutation	23	Ion AmpliSeq Colon and Lung Cancer Research (v2), etc
Patil et al.^[14]^	HER2 mutation	29	Sanger sequencing, NGS, etc
	HER2 amplification	2	FISH (HER2/CEP17≥3)
Chu et al.^[15]^	HER2 mutation	26	ARMS-PCR, DNA direct sequencing
van Berge Henegouwen et al.^[16]^	HER2 mutation	24	NGS
Mazières et al.^[17]^	HER2 mutation	101	Direct sequencing after PCR amplification, NGS
Kinoshita et al.^[18]^	HER2 mutation or overexpression	10	-
Hainsworth et al.^[19]^	HER2 mutation	14	NGS
	HER2 amplification or overexpression	16	HER2 amplification: NGS, FISH/CISH (HER2/CEP17>2.0 or copy number>6); HER2 overexpression: IHC3+
Li et al.^[20]^	HER2 mutation	18	NGS
Iwama et al.^[21]^	HER2 mutation	22	NGS, multiplex real-time PCR
Li et al.^[22] ^	HER2 mutation	28	NGS
	HER2 amplification	11	NGS, FISH
Peters et al.^[23]^	HER2 overexpression	49	IHC（IHC2+/3+）
Tsurutani et al.^[24]^	HER2 mutation	11	NGS, etc
Nakagawa et al.^[25]^	HER2 overexpression	49	IHC (IHC2+/3+)
Goto et al.^[26] ^	HER2 mutation	PEC: 80	-
		SAS: 151	
Song et al.^[27] ^	HER2 mutation	54	NGS
	HER2 amplification	12	NGS
Lai et al.^[28]^	HER2 mutation	23	RT-PCR, NGS
Fan et al.^[29]^	HER2 mutation	18	AmoyDx® HER2 mutation detection kit
Dziadziuszko et al.^[30]^	HER2 mutation	13	PCR, NGS, etc
Peters et al.^[31]^	HER2 mutation	28	-
Yang et al.^[32]^	HER2 mutation	172	NGS
Kris et al.^[33]^	HER2 mutation	26	-
	HER2 amplification	4	FISH (HER2/CEP17≥2)
Robichaux et al.^[34]^	HER2 mutation	12	NGS
Prelaj et al.^[35]^	HER2 mutation	8	NGS, Sanger sequencing, etc
Elamin et al.^[36] ^	HER2 mutation	30	Oncomine Comprehensive Assay, Guardant360 Assay (using plasma), FoundationOne Assay
Le et al.^[37]^	HER2 mutation	90	-
Wang et al.^[38] ^	HER2 mutation	15	NGS, direct Sequencing, etc
Zhou et al.^[39] ^	HER2 mutation	60	ADx HER2 mutation detection kit
Song et al.^[40]^	HER2 mutation	78	NGS
Song et al.^[41]^	HER2 amplification	27	NGS
Yang et al.^[42]^	HER2 mutation	31	NGS, ARMS-PCR
	HER2 amplification	2	NGS, ARMS-PCR
Liu et al.^[43]^	HER2 mutation	11	-

HER2: human epidermal growth factor receptor 2; NSCLC: non-small cell lung cancer; ARMS-PCR: amplification refractory mutation system polymerase chain reaction; NGS: next generation sequencing; FISH: fluorescence in situ hybridization; CEP17: centromere of chromosome 17; CISH: chromogenic in situ hybridization; IHC: Immunohistochemistry; RT-PCR: reverse transcription-PCR; PEC: prespecified early cohort; SAS: safety analysis set.

### 2.1 HER2阳性NSCLC检测样本的制作

肿瘤组织、循环肿瘤DNA（circulating tumor DNA, ctDNA）、细胞学标本均可作为HER2阳性NSCLC的检测样本。其中，肿瘤组织是首选检测样本。制作组织标本时应注意以下几点：（1）获得肿瘤组织后应尽快在1 h内将其固定或在10 min内用液氮保存。（2）组织标本应按5 mm-10 mm的厚度进行切片，并在10%中性缓冲福尔马林中固定6 h-72 h。（3）为防止抗原丢失，未染色的切片不应在室温下放置超过6周^[44,45]^。

在肿瘤组织标本不足或无法获得组织标本的情况下，可使用血液样本进行检测。外周血ctDNA采集时应注意以下几点：（1）使用一次性封闭式乙二胺四乙酸抗凝真空采血管采样；若样本处理前需在室温下放置一定时间，也可以使用Streck管或其他细胞游离DNA收集管。（2）最少采集6 mL-10 mL全血，采用双链分离技术分离血浆，并在6 h内提取ctDNA。（3）ctDNA应在-80 ^o^C下储存，并应避免重复解冻^[45,46]^。

### 2.2 HER2突变的诊断

美国食品药品监督管理局（Food and Drug Administration, FDA）已批准Trastuzumab Deruxtecan（T-DXd，曾用名：DS-8201）用于治疗HER2突变的无法切除或转移性NSCLC患者。美国国立综合癌症网络（National Comprehensive Cancer Network, NCCN）指南也已将HER2突变列入NSCLC推荐基因检测列表中。HER2突变已成为NSCLC靶向治疗的重要靶点。

HER2突变常发生在TKD的外显子18-21中，其中以外显子20的插入突变最为常见。外显子20插入突变的范围为3-12碱基对（base pair, bp），都嵌套在外显子的最近端，密码子775-881之间，其中又以第775密码子处12 bp（编码YVMA）帧内重复插入最为常见，即最常见的HER2外显子20插入突变亚型是A775_G776insYVMA。其次为G778_P780dup和G776delinsVC。TKD的突变导致腺苷三磷酸（adenosine triphosphate, ATP）结合口袋的构象改变，从而增强激酶活性和下游信号传导，最终导致肿瘤的发生发展^[6⇓⇓-9,47]^。

目前检测HER2突变的方法有Sanger测序、下一代测序（next generation sequencing, NGS）、扩增难治性突变系统聚合酶链反应（amplification refractory mutation system polymerase chain reaction, ARMS-PCR）和液滴数字PCR（droplet digital PCR, ddPCR）等（表1^[2,9⇓⇓⇓⇓⇓⇓⇓⇓⇓⇓⇓⇓⇓⇓⇓⇓⇓⇓⇓⇓⇓⇓⇓⇓⇓⇓⇓⇓⇓⇓⇓⇓⇓-43]^）。2022年发表的一篇专家共识^[45]^中推荐优先使用肿瘤组织进行NGS检测来诊断HER2突变。此外，样本中应有足够比例的肿瘤细胞，这是决定检测结果可靠性的关键。NGS组织样本中的最佳肿瘤细胞含量为40%，最低肿瘤细胞含量应在10%-20%之间。

### 2.3 HER2扩增的诊断

NGS、实时定量逆转录酶PCR（real-time quantitative reverse transcriptase polymerase chain reaction, qRT-PCR）和荧光原位杂交（fluorescence in situ hybridization, FISH）技术均可用于检测HER2扩增（表1^[2,9⇓⇓⇓⇓⇓⇓⇓⇓⇓⇓⇓⇓⇓⇓⇓⇓⇓⇓⇓⇓⇓⇓⇓⇓⇓⇓⇓⇓⇓⇓⇓⇓⇓-43]^）。临床上，检测NSCLC中HER2扩增常用的方法是NGS。FISH可测定HER2基因和17号染色体着丝粒（centromere of chromosome 17, CEP17）的拷贝数变化，因此，也被推荐用于HER2扩增的检测。目前推荐NSCLC中HER2扩增的标准为：HER2/CEP17≥2.0或HER2拷贝数≥6.0或多个HER2信号聚集^[45,48]^。但也有学者认为，HER2/CEP17<2.0但每个细胞HER2基因拷贝数≥6.0为HER2拷贝数增加（copy number gain, CNG）的表现，应该将HER2扩增和HER2 CNG两者区别开来。HER2 CNG是17号染色体多聚体的结果。17号染色体多聚体导致每个细胞HER2拷贝数增加，从而提高了上游启动子的活性，导致HER2基因表达量增加。因此，HER2/CEP17≥2.0被认为更能反映HER2的扩增^[8]^。此外，部分学者认为血液数字PCR也可能是检测NSCLC HER2扩增的潜在方法^[49]^。

### 2.4 HER2过表达的诊断

HER2过表达通常通过免疫组化（immunohistochemistry, IHC）来诊断（表1^[2,9⇓⇓⇓⇓⇓⇓⇓⇓⇓⇓⇓⇓⇓⇓⇓⇓⇓⇓⇓⇓⇓⇓⇓⇓⇓⇓⇓⇓⇓⇓⇓⇓⇓-43]^）。有学者^[50]^简单地评估了NSCLC中HER2染色模式及其与FISH结果的相关性，他们用乳腺癌和胃癌中HER2阳性的标准去评估每个福尔马林固定石蜡包埋样品中HER2阳性模式的特征，结果表明，相较于乳腺癌，胃癌的HER2阳性标准更适用于NSCLC，即更推荐NSCLC的HER2 IHC评分标准为：HER2 IHC0、IHC1+定义为HER2阴性；若≥10%的肿瘤细胞呈弱至中等染色则定义为IHC2+；若≥10%的肿瘤细胞呈强完全、基底外侧或外侧膜染色则定义为IHC3+。有学者^[51]^认为在HER2 IHC2+或IHC3+的情况下，需要执行额外的原位杂交分析来与HER2扩增、多体性等相鉴别。也有学者^[45]^参考乳腺癌中的IHC评分，但认为相较于乳腺癌，NSCLC中HER2扩增与过表达之间没有明显的相关性，且NSCLC中FISH和IHC的一致性较差，建议IHC2+、IHC3+即确认为HER2过表达，无需FISH再确认HER2表达。除IHC和FISH外，实时荧光定量PCR和qRT-PCR等也可定量检测HER2基因过表达^[51]^。

## 3 HER2阳性NSCLC的治疗进展

### 3.1 化疗

近年来关于化疗在HER2阳性NSCLC患者中疗效的研究较少。在一项回顾性研究^[10]^中，接受一线化疗的HER2突变肺癌患者的客观缓解率（objective response rate, ORR）为6.9%，中位无进展生存期（progression-free survival, PFS）为5.9个月。在另一项研究^[11]^中，研究者们比较了HER2突变、EGFR突变、间变性淋巴瘤激酶（anaplastic lymphoma kinase, ALK）/c-ros致癌基因1受体酪氨酸激酶（ROS proto-oncogene 1 receptor tyrosine kinase, ROS1）重排和Kirsten鼠类肉瘤（kirsten rat sarcoma, KRAS）突变晚期肺腺癌患者基于培美曲塞一线化疗的疗效。在该研究中，HER2突变组患者的ORR为36.0%，中位PFS为5.1个月；EGFR突变组、ALK/ROS1重排组和KRAS突变组的中位PFS分别为6.5个月、9.2个月和5个月，ORR则与HER2突变组相比无显著差异。该研究结果表明，相较于ALK/ROS1重排患者，晚期HER2突变肺腺癌患者对基于培美曲塞一线化疗的反应较差，这进一步加强了临床实践中对有效HER2靶向药物的需求。此外，一项回顾性研究^[9]^表明，与单独化疗相比，化疗联合血管生成抑制剂可能会对HER2阳性肺癌患者产生更大的生存益处（中位PFS：4.03个月 vs 5.63个月）。

从现有研究结果来看，HER2突变NSCLC对化疗的反应可能较其他驱动基因阳性NSCLC差。同时，与单独化疗相比，联合抗血管生成药物治疗似乎对于HER2阳性NSCLC更加优越。

### 3.2 免疫治疗

多项肺癌临床试验已证明抗程序性死亡受体1（programmed cell death 1, PD-1）或抗程序性死亡配体1（programmed cell death ligand 1, PD-L1）免疫疗法优于化疗，也已经被推荐为IV期NSCLC患者的一线或二线治疗方案^[52]^。但其在HER2阳性NSCLC中的疗效研究有限且结论不一。多项回顾性研究^[12,13]^表明HER2突变NSCLC患者并未在ICIs或ICIs联合铂类化疗中明显获益。在法国的一项回顾性研究^[13]^中，23例HER2突变NSCLC患者接受单药ICIs治疗，ORR为27.3%，中位PFS为2.2个月，中位总生存期（overall survival, OS）为20.4个月。也有研究^[14]^表明即使具有高PD-L1表达，HER2扩增或突变患者对单药ICIs的反应也较差。然而，另一项回顾性研究^[15]^表明，ICIs对HER2突变肺癌患者有较好的疗效。该研究纳入了26例患者，其ORR为38.5%，疾病控制率（disease control rate, DCR）为84.6%，中位PFS为7.4个月。该项研究中61.5%的患者接受了免疫联合化疗方案治疗，这些患者的中位PFS为8.4个月。然而这些研究都是回顾性研究，关于免疫治疗在HER2阳性NSCLC患者中的疗效，仍需要更大样本的前瞻性研究来验证。另有一项临床前研究^[53]^表明，T-DXd和PD-1抗体的联合治疗可能比任何一种单一治疗都更有效。

综上，ICIs单一疗法对HER2阳性的NSCLC患者疗效可能是有限的；但ICIs联合其他药物是否可增加疗效，仍需要继续被研究；ICIs联合靶向药物疗法似乎有较大的前景，这可能是HER2阳性NSCLC未来的研究领域。

### 3.3 靶向治疗

#### 3.3.1 抗体

HER2阳性乳腺癌靶向治疗的成功极大地影响了HER2阳性NSCLC的治疗发展。曲妥珠单抗治疗方案是HER2阳性乳腺癌的标准治疗方案。曲妥珠单抗是一种重组人源单克隆抗体，能够结合HER2的ECD，从而阻断HER2同二聚或与HER3配体无关的异二聚，抑制致癌信号传导，然而，曲妥珠单抗不能阻止HER2与其他HER分子的配体依赖性异源二聚，这可能会导致肿瘤细胞逃逸^[16,54]^（表2^[16⇓⇓-19]^）。一项欧洲的回顾性研究^[17]^报告了HER2突变NSCLC患者可能对曲妥珠单抗和化疗的组合特别敏感，该研究中曲妥珠单抗联合化疗组的ORR高达50%。然而，一项II期试验^[18]^结果显示，HER2阳性的NSCLC患者对曲妥珠单抗单药治疗并未产生应答。该试验招募了10例HER2阳性肺癌患者，尽管该试验的DCR为70%，中位PFS为5.2个月，但ORR为0%。但该试验样本量较小，其得出的结论值得怀疑，关于曲妥珠单抗单药对HER2阳性肺癌疗效的研究仍需要更大型的前瞻性随机试验来证明。

**表2:** 单克隆抗体在HER2阳性NSCLC中的疗效

Author	Drug	Study type	HER2 status	n	ORR	mPFS (mon)	mOS (mon)
van Berge Henegouwen et al.^[16]^	Trastuzumab+ Pertuzumab	II	Mutation	24	8.3%	4.0	10.0
Mazières et al.^[17]^	Trastuzumab/T-DM1+ chemotherapy	Retrospective study	Mutation	58	50.9%	4.8	13.3
Kinoshita et al.^[18]^	Trastuzumab	II	Mutation or overexpression	10	0.0%	5.2	-
Hainsworth et al.^[19]^	Trastuzumab+ Pertuzumab	IIa	Mutation	14	21.0%	-	-
			Amplification or overexpression	16	13.0%	-	-

ORR: objective response rate; mPFS: median progression-free survival; mOS: median overall survival; T-DM1: Trastuzumab Emtansine; IHC: Immunohistochemistry.

帕妥珠单抗是另一种抗HER2单克隆抗体，是一种HER异源二聚体抑制剂，抑制HER2与其他HER分子的异源二聚以及配体诱导的与HER3的二聚，当其与曲妥珠单抗联合时可提供更完整的HER2下游信号双重阻断，从而抑制癌症的发生^[16]^。在一项II期试验^[19]^中，14例HER2突变NSCLC患者接受了曲妥珠单抗联合帕妥珠单抗治疗，ORR为21%；另有16例HER2扩增或过表达患者接受相同治疗后，ORR为13%。在另一项研究^[16]^中，24例HER2外显子20突变NSCLC患者接受了曲妥珠单抗联合帕妥珠单抗治疗，在38%的患者中观察到了临床获益，ORR为8.3%。尽管该研究达到了其主要疗效终点——临床获益；但曲妥珠单抗联合帕妥珠单抗仅在部分接受过重度预处理的HER2外显子20突变患者中略有活性。

总体而言，与HER2阳性乳腺癌相比，单克隆抗体在HER2阳性NSCLC中的疗效有限。尽管曲妥珠单抗联合化疗在HER2突变NSCLC患者中有较好的ORR，但仍需要进一步研究以比较联合化疗和单独化疗的疗效。

#### 3.3.2 抗体药物偶联物

Trastuzumab Emtansine（T-DM1）是一种由曲妥珠单抗和细胞毒性微管抑制剂Emtansine（DM1）通过稳定接头连接的ADCs，其通过受体介导的内吞作用渗透到HER2阳性细胞中，并在溶酶体中发生降解释放DM1，DM1能有效抑制微管蛋白聚合，进而阻滞细胞有丝分裂，导致肿瘤细胞凋亡^[55]^（表3^[2,20⇓⇓⇓⇓⇓-26]^）。一项研究T-DM1对HER2突变型肺腺癌疗效的II期试验^[20]^纳入了18例患者，ORR为44%，达到了该试验的主要终点，中位PFS为5个月。其中11例HER2外显子20插入突变患者的ORR明显高于其余7例其他HER2突变患者（55% vs 29%）。另有一项研究T-DM1疗效的II期试验^[21]^纳入了22例HER2外显子20插入突变患者，ORR为38.1%，中位PFS为2.8个月，中位OS为8.1个月。尽管该试验中T-DM1对HER2外显子20插入突变患者疗效显著，但近一半的患者对该药物没有任何反应，这可能是中位PFS相对较低的原因。这些研究表明，T-DM1可能是HER2突变肺癌患者的潜在治疗药物，但仍需进一步研究T-DM1治疗的潜在生物标志物。关于T-DM1在HER2扩增患者中的疗效也已在一项II期试验中被证明，55%的HER2扩增患者接受该药治疗后得到了客观缓解，这表明HER2扩增可能是T-DM1潜在生物标志物^[22]^。Peters等^[23]^研究分析了T-DM1对HER2过表达（IHC2+或IHC3+）NSCLC患者的疗效。该研究纳入了49例患者（29例IHC2+，20例IHC3+），在IHC2+队列中未观察到治疗反应；而在IHC3+队列中观察到ORR为20%（4/20），然而在这4例应答者中，3例患者伴有HER2扩增，2例患者伴有HER2突变；在任一队列中均未观察到PFS或OS优势（中位PFS：2.6个月 vs 2.7个月；中位OS：12.2个月 vs 15.3个月），因此，HER2过表达可能并不是T-DM1的潜在生物标志物。

**表3:** ADCs在HER2阳性NSCLC中的疗效

Author	Drug	Study type	HER2 status	n	ORR	mPFS (mon)	mOS (mon)
Li et al.^[2]^	T-DXd	II	Mutation	91	55.0%	8.2	17.8
Li et al.^[20]^	T-DM1	II	Mutation	18	44.0%	5.0	-
Iwama et al.^[21]^	T-DM1	II	Mutation	22	38.1%	2.8	8.1
Li et al.^[22]^	T-DM1	II	Positive	49 (Total)	51.0%	5.0	-
			Mutation	28	50.0%	-	-
			Amplification	11	55.0%	-	-
			Mutation combined amplification	10	50.0%	-	-
Peters et al.^[23]^	T-DM1	II	Overexpression	49 (Total)	-	-	-
				29 IHC2+	0.0%	2.6	12.2
				20 IHC3+	20.0%	2.7	15.3
Tsurutani et al.^[24]^	T-DXd	I	Mutation	11	72.7%	11.3	-
Nakagawa et al.^[25]^	T-DXd	II	Overexpression	49	24.5%	5.4	-
Goto et al.^[26]^	T-DXd	II	Mutation	80 (Total)	-	-	-
				52 (5.4 mg/kg)	53.8%	-	-
				28 (6.4 mg/kg)	42.9%	-	-

ADC: antibody-drug conjugate; T-DXd: Trastuzumab Deruxtecan.

综上，T-DM1在HER2过表达NSCLC患者中的疗效未达到预期效果，但其在HER2突变和扩增的NSCLC患者中具有一定的临床疗效，目前仍需进一步研究T-DM1在NSCLC中的生物标志物。

T-DXd由曲妥珠单抗和拓扑异构酶I抑制剂Deruxtecan（DXd）组成，两者通过基于四肽的可裂解接头连接。当T-DXd进入肿瘤细胞后，四肽键被溶酶体水解，从而释放DXd，DXd可结合拓扑异构酶I-DNA复合物，从而诱导DNA双链断裂，最终诱导肿瘤细胞凋亡^[56]^（表3^[2,20⇓⇓⇓⇓⇓-26]^）。在一项I期研究^[24]^中，T-DXd在HER2突变NSCLC的患者群体中显示出较好的活性，ORR为72.7%（8/11），中位PFS为11.3个月。但该研究数量有限，T-DXd的疗效需要在更大的研究中得到证实。DESTINY-Lung01是一项研究T-DXd在HER2突变以及HER2过表达（IHC2+/3+）NSCLC患者中疗效和安全性的II期研究^[2]^。在该研究中，91例HER2突变NSCLC患者每3周给予6.4 mg/kg的T-DXd，结果显示T-DXd在这些患者中有持久的抗癌活性：ORR为55%，中位PFS为8.2个月，中位OS为17.8个月。但是在该研究中，49%的患者发生了≥3级治疗相关不良反应（treatment related adverse events, TRAEs）；26%的患者发生了药物相关间质性肺病；由于TRAEs而导致永久停药、减量和暂停用药的患者比例分别为25%、34%和32%，因此T-DXd的安全性问题值得重视。在49例接受同样治疗的HER2过表达（IHC2+/3+）NSCLC患者中，24.5%的患者得到了客观缓解，中位PFS为5.4个月，其中，IHC2+和IHC3+患者的ORR分别为25.6%和20.0%，并无差异^[25]^。可见，T-DXd在HER2过表达NSCLC患者中的疗效并不如其在HER2突变NSCLC患者中的疗效。虽然T-DXd在HER2突变NSCLC患者中具有显著前景，但其带来的不良反应不容小觑。为了探索T-DXd降低剂量后，是否可以保持疗效并降低TRAEs发生率，研究者们进行了DESTINY-Lung02试验^[26]^。该试验研究了T-DXd 5.4 mg/kg和6.4 mg/kg在HER2突变患者中的疗效和安全性。其中期结果显示，在预先设定的早期队列中，5.4 mg/kg组的ORR可达53.8%，6.4 mg/kg组的ORR为42.9%；在安全性分析组中，T-DXd 5.4 mg/kg组患者≥3级TRAEs发生率为31.7%，间质性肺炎发生率仅为5.9%；T-DXd 6.4 mg/kg组患者≥3级TRAEs发生率为58%，间质性肺炎发生率为14.0%。显而易见，5.4 mg/kg剂量的T-DXd在HER2突变患者中也有较好的疗效，且安全性更好^[26]^。

综上，T-DXd在HER2突变的NSCLC中具有显著的前景，在HER2过表达的NSCLC中的疗效有限。基于DESTINY-Lung02研究，FDA已经批准了T-DXd用于治疗不可切除或转移性HER2突变NSCLC的成年患者。NCCN指南也将T-DXd列入了HER2突变NSCLC的推荐用药。2023年2月24日，T-DXd在国内获批上市，用于治疗既往接受过一种或一种以上抗HER2药物治疗的不可切除或转移性HER2阳性成人乳腺癌患者。目前关于T-DXd在HER2阳性NSCLC的相关研究仍在进行中，T-DXd与其他药物联合治疗的研究成果也非常令人期待。

在HER2阳性NSCLC中，ADCs是目前最具有前景的药物，除T-DM1和T-DXd这两种ADCs外，其他ADCs药物，如A166、XMT-1522等也都正在被研究^[57]^。

#### 3.3.3 TKIs

非选择性TKIs在HER2阳性NSCLC患者中显示出的抗肿瘤活性有限（表4^[27⇓⇓⇓⇓⇓⇓⇓⇓⇓⇓⇓⇓⇓⇓⇓-43]^）。

**表4:** TKIs在HER2阳性NSCLC中的疗效

Author	Drug	Study type	HER2 status	n	ORR	mPFS (mon)	mOS (mon)
Song et al.^[27]^	Afatinib	Retrospective study	Positive	66 (Total)	24.0%	3.3	13.9
			Mutation	54	22.0%	3.4	14.6
			Amplification	12	33.0%	3.3	13.4
Lai et al.^[28]^	Afatinib	Retrospective study	Mutation	23	13.0%	-	7.0
Fan et al.^[29]^	Afatinib	II	Mutation	18	0.0%	2.8	10.0
Dziadziuszko et al.^[30]^	Afatinib	II	Mutation	13	7.7%	3.7	13.1
Peters et al. ^[31]^	Afatinib	II	Mutation	28	19.0％	-	-
Yang et al.^[32]^	Afatinib	Retrospective study	Mutation	26 (First-line)	11.5%	3.4	-
			Mutation	24 (Second-line)	8.3%	2.9	-
	Pyrotinib	Retrospective study	Mutation	9 (First-line)	22.2%	6.8	-
			Mutation	27 (Second-line)	14.8%	5.8	-
Kris et al.^[33]^	Dacomitinib	II	Mutation	26	12.0%	3.0	9.0
			Amplification	4	0.0%	-	-
Robichaux et al.^[34]^	Poziotinib	II	Mutation	12	42.0%	5.6	-
Prelaj et al.^[35]^	Poziotinib	II	Mutation	8	50.0%	-	-
Elamin et al.^[36]^	Poziotinib	II	Mutation	30	27.0%	5.5	15.0
Le et al.^[37]^	Poziotinib	II	Mutation	90	27.8%	5.5	-
Wang et al.^[38]^	Pyrotinib	II	Mutation	15	53.3%	6.4	12.9
Zhou et al.^[39]^	Pyrotinib	II	Mutation	60	30.0%	6.9	14.4
Song et al.^[40]^	Pyrotinib	II	Mutation	78	19.2%	5.6	10.5
Song et al.^[41]^	Pyrotinib	II	Amplification	27	22.2%	6.3	12.5
Yang et al.^[42]^	Pyrotinib+ Apatinib	II	Mutation or amplification	33	51.5%	6.9	14.8
Liu et al.^[43]^	Tarloxotinib	II	Mutation (cohort B)	11	22.2%	-	-

TKIs: tyrosine kinase inhibitors.

非选择性TKIs阿法替尼已被批准用于EGFR突变的NSCLC患者，但其在HER2突变的NSCLC患者中的疗效有待商榷。一项国内回顾性研究^[27]^表明，阿法替尼在54例HER2突变肺癌患者中的ORR为22%，中位PFS为3.4个月，中位OS为14.6个月。该试验也进一步比较了42例HER2外显子20突变患者和12例HER2其他突变患者的预后，结果显示HER2外显子20突变患者的预后比其他HER2突变患者差（ORR：17% vs 42%；中位PFS：2.6个月 vs 5.8个月；中位OS：12.9个月 vs 33.3个月）。在一项国际性的回顾性研究^[28]^中，阿法替尼在23例HER2突变肺腺癌患者中的ORR为13%，中位OS为7个月，在该研究中，6例患者因TRAEs而停止治疗，常见的TRAEs主要是腹泻。而一项面向亚洲HER2突变NSCLC患者的研究结果^[29]^显示，阿法替尼对EGFR-TKIs初治的HER2突变NSCLC患者没有临床益处，该研究中接受阿法替尼治疗的18例患者均没有达到客观缓解。此外，一项II期试验^[30]^纳入了13例HER2突变NSCLC患者，只有1例患者接受阿法替尼治疗后出现了客观缓解，中位PFS和中位OS分别为15.9周和56.0周。阿法替尼在HER2突变NSCLC患者中的疗效不同，可能与HER2突变类型不同有关。有研究^[31]^表明HER2外显子20插入A775_G776insYVMA突变似乎对阿法替尼比较敏感，10例HER2外显子20插入A775_G776insYVMA突变患者中，有4例患者接受阿法替尼治疗长达1年以上，ORR为33%，DCR为100%。然而在另一项研究^[32]^中，阿法替尼似乎对HER2外显子20插入G776delinsVC突变患者更为敏感，ORR为20.0%-28.6%，中位PFS为4.3个月-7个月；在HER2外显子20插入A775_G776insYVMA突变患者中，ORR则为0%-9.5%，中位PFS则为2.8个月-3.2个月。为了研究阿法替尼在HER2扩增NSCLC患者中的疗效，一项研究^[27]^纳入了12例HER2扩增NSCLC患者，结果显示阿法替尼的ORR为33%，中位PFS为3.3个月。关于阿法替尼在HER2扩增及过表达NSCLC患者中的治疗研究仍需要进一步开展。

达可替尼和奈拉替尼也是两种非选择性TKIs，两者均可与EGFR、HER2以及HER4不可逆结合。一项II期试验纳入了26例HER2突变NSCLC患者和4例HER2扩增NSCLC患者，达可替尼在其中的ORR分别为12%和0%，在HER2突变患者中的中位PFS为3个月，中位OS为9个月。达可替尼最常见的TRAEs为腹泻（90%）和皮疹（73%）^[33]^。近年来，也有学者研究了奈拉替尼在HER2阳性NSCLC细胞中的抗肿瘤作用，他们发现奈拉替尼可以抑制HER2和EGFR的磷酸化，并能抑制HER2扩增和HER2突变肺癌细胞的下游信号传导。由此可见，奈拉替尼在HER2阳性NSCLC中可能有一定的前景^[58]^。

总体而言，与ADCs相比，阿法替尼、达可替尼、奈拉替尼等选择性较低的TKIs对HER2阳性的NSCLC的疗效并不理想。相较于这些低选择性TKIs，新型TKIs更有利于HER2阳性的NSCLC患者（表4^[27⇓⇓⇓⇓⇓⇓⇓⇓⇓⇓⇓⇓⇓⇓⇓-43]^）。

波齐替尼具有体积小、结构灵活等特点，使得其可规避HER2外显子20插入突变在药物结合袋中的空间位阻，从而发挥抗肿瘤作用。多项研究^[34,59,60]^表明，与阿法替尼、达可替尼、奈拉替尼等多种TKIs相比，波齐替尼对HER2突变型NSCLC的抑制作用更强，此外，波齐替尼可上调HER2细胞表面表达并增强T-DM1的活性，从而通过联合治疗使肿瘤完全消退。一项II期临床研究^[34]^中，波齐替尼在12例HER2外显子20突变NSCLC患者中的ORR为42%，中位PFS为5.6个月。该研究中波齐替尼的TRAEs与阿法替尼、达可替尼等非选择性TKIs相似，8例患者出现了3级-4级TRAEs，以腹泻（17%）和皮疹（58%）最为多见。在一项纳入了30例EGFR或HER2外显子20插入突变患者的研究^[35]^中，所有患者的中位PFS为5.6个月，HER2亚组的ORR为50%（4/8）。另一项研究^[36]^中，27%的HER2突变NSCLC患者接受波齐替尼治疗后得到了客观缓解，中位PFS为5.5个月。97%的患者出现了TRAEs，大多是皮疹（83%）、腹泻（80%）、甲沟炎（70%）、口腔黏膜炎（67%）和皮肤干燥（63%）；1例患者（3%）因TRAEs而停用波齐替尼，16例患者（53%）减少了一次剂量，6例患者（20%）减少了两次剂量。在ZENITH20-2研究^[37]^中，波齐替尼在90例既往接受过治疗的HER2突变NSCLC患者中显示出抗肿瘤活性（ORR：27.8%，中位PFS：5.5个月）。然而该研究中，88例患者发生了TRAEs，其中71例患者发生了3级TRAEs，4例患者发生了4级TRAEs，以皮疹、腹泻、口腔炎多见。只有21例患者在整个研究过程中没有减量（16 mg, qd），12例患者因TRAEs而停止治疗。尽管波齐替尼在HER2突变患者中有一定的疗效，但其所带来的安全性问题限制了它在HER2突变NSCLC患者中的应用，波齐替尼的合适治疗方案仍需探索。

吡咯替尼，相较于阿法替尼和T-DM1，在插入HER2-A775_G776YVMA人源肿瘤异种移植模型中，显示出更强的抗肿瘤效果。研究者们在15例HER2突变患者中进行了一项II期临床试验^[38]^，结果表明，吡咯替尼的ORR为53.3%，中位PFS为6.4个月；其中10例HER2 A775_G776insYVMA插入突变患者的ORR为50%，中位PFS为4.1个月。在一项纳入了60例HER2突变NSCLC患者的II期试验^[39]^中，吡咯替尼的ORR为30%，中位PFS为6.9个月。98.3%的患者发生了TRAEs，最为常见的是腹泻（91.7%），17例患者发生了3级-4级TRAEs。因TRAEs而中断或停止治疗的患者比例分别为1.7%和21.7%。此外，还有多项研究^[32,40]^均表明吡咯替尼在HER2突变肺癌患者中有良好的抗肿瘤活性。吡咯替尼在HER2扩增患者中的疗效，也在相关研究中得到了证实。在27例HER2扩增的队列中，观察到吡咯替尼的ORR为22.2%，中位PFS为6.3个月。所有患者均发生了TRAEs（3级：22.2%），最常见的TRAEs为腹泻（92.6%）^[41]^。PATHER2是一项研究吡咯替尼联合抗血管生成药物阿帕替尼在HER2突变或扩增NSCLC患者中的疗效的II期试验^[42]^。这项试验的ORR为51.5%，中位PFS为6.9个月，所有患者均发生了TRAEs，最常见的是腹泻（90.9%）、高血压（72.7%）、厌食（54.5%）、恶心（51.5%）和口腔黏膜炎（45.5%）。可见，吡咯替尼联合阿帕替尼在HER2阳性的肺癌患者中也具有一定的治疗前景。然而，同波齐替尼一样，吡咯替尼所带来的安全性问题也阻碍了其在HER2阳性肺癌患者中的应用。

溴他替尼是一种新型的缺氧活化前药，可在肿瘤缺氧微环境中转化成有活性的溴他替尼效应物。溴他替尼在HER2突变的NSCLC中已显示出临床前疗效^[61]^。RAIN-701试验^[43]^中，22%（2/9）的HER2突变患者接受溴他替尼治疗后出现了客观缓解。

2023年1月，莫博赛替尼获得国家药品监督管理局批准正式进入中国。莫博赛替尼是全球首款也是目前唯一获批的治疗EGFR外显子20插入突变晚期NSCLC的口服靶向药物。莫博赛替尼也在临床前研究中被证明对HER2外显子20插入突变NSCLC有一定的抗肿瘤活性，且与T-DM1联用时有一定的协同作用^[62]^。其在HER2突变NSCLC患者中的临床疗效仍需进一步研究。

综上，新型TKIs在HER2阳性的NSCLC中具有较好的抗肿瘤活性，但其所带来的药物不良反应限制了其应用，目前仍需进一步研究来改善新型TKIs的临床疗效和毒性管理。其他新型TKIs药物的研发试验目前也正在进行。

## 4 小结和展望

随着分子生物学技术的发展，NSCLC中HER2突变、扩增、过表达的检测手段越来越多样。这在一定程度上推动了HER2阳性NSCLC的治疗研究。HER2突变是目前研究相对充分的生物标志物，HER2扩增和过表达的诊断标准仍需要进一步确立，以便更好地开展相应治疗研究，制定相应的治疗方案以及明确HER2突变、扩增、过表达三者之间的关系。ADCs和新型TKIs在HER2阳性NSCLC中显示出较好的临床活性，这为HER2阳性NSCLC的靶向治疗提供了希望。虽然目前化疗、ICIs单一疗法在HER2阳性NSCLC中的疗效不如ADCs和新型TKIs，但靶向疗法与化疗、免疫治疗或抗血管生成药物的联合治疗是否会有更好的临床疗效，这仍需进一步探索。

为了提高抗HER2药物的疗效，HER2阳性NSCLC的联合治疗模式正在被研究。例如，DESTINY-Lung03旨在研究T-DXd联合度伐利尤单抗和化疗在HER2阳性NSCLC中的疗效和安全性；NCT04042701旨在研究T-DXd联合帕博利珠单抗在HER2阳性NSCLC中的疗效；NCT05378763旨在研究波齐替尼在铂类化疗后的作用^[63,64]^。此外，一些临床试验也正在积极研究新型TKIs，如BDTX-189、AST2818、DZD9008和BAY2927088等^[63]^。随着研究的深入，HER2阳性NSCLC的诊疗体系将会越来越完善。
